# Burden, satisfaction caregiving, and family relations in informal caregivers of older adults

**DOI:** 10.3389/fmed.2022.1059467

**Published:** 2022-12-22

**Authors:** Jéssica da Silva Marinho, Ilaise Brilhante Batista, Rute Alessandra da Silva Nobre, Maria Sortênia Alves Guimarães, Ariene Angelini dos Santos-Orlandi, Tábatta Renata Pereira Brito, Valéria Pagotto, Maria Giovana Borges Saidel, Suzimar de Fátima Benato Fusco, Flavia de Oliveira Motta Maia, Ligiana Pires Corona, Daniella Pires Nunes

**Affiliations:** ^1^Postgraduate Science and Health Teaching Program, Federal University of Tocantins, Palmas, Tocantins, Brazil; ^2^Graduate Program in Nursing, University of Campinas, São Paulo, Brazil; ^3^Department of Medicine, Federal University of Tocantins, Palmas, Tocantins, Brazil; ^4^Department of Nursing, Federal University of São Carlos, São Paulo, Brazil; ^5^School of Nutrition, Federal University of Alfenas, Alfenas, Minas Gerais, Brazil; ^6^School of Nursing, Federal University of Goiás, Goiânia, Goiás, Brazil; ^7^School of Nursing, University of Campinas, São Paulo, Brazil; ^8^School of Applied Sciences, University of Campinas, São Paulo, Brazil

**Keywords:** caregivers, caregiver burden, frail elderly, family relations, family

## Abstract

**Introduction:**

Family caregivers of older persons devote much of their time and energy to caring for another person. This exposure may burden caregivers and compromise their health and quality of life.

**Objective:**

To investigate the relationship between burden, sociodemographic, caregiving, and health characteristics of informal caregivers of dependent older adults.

**Methods:**

Cross-sectional and analytical study carried out in Palmas, Tocantins, Brazil, with 52 informal caregivers of older persons who need full-time help for basic living activities. Caregivers' burden was assessed by Zarit Burden Interview (ZBI). Data were analyzed using a *T*-test, Pearson's correlation, and Multiple Linear Regression.

**Results:**

The ZBI mean score of caregivers was 26.3 points (SD = 14.6; min = 0; max = 68). Burden scores were higher among caregivers who did not receive help from other people in care (*p* = 0.016), reported family dysfunction (*p* = 0.001), and had depression symptoms (*p* = 0.007). A correlation was found between the scores of burdens and satisfaction with care (*r* = 0.76; *p* < 0.001) and perceived material support (*r* = −0.30; *p* = 0.40). Satisfaction with care (β: 0.61; *p* < 0.001) and family dysfunction (β: 8.07; *p* = 0.033) were significantly associated with the burden score.

**Conclusion:**

Caregivers with dysfunctional families and satisfaction with the care presented the highest-burden scores. The findings reveal the need for strategies to facilitate mediation and reduce caregiver burden by strengthening the family network support or providing professional assistance.

## 1. Introduction

Care is a human need. Humans give and receive care throughout life. The demand for care is associated with the functional impairment of older adults; that is, the person requires help from someone to perform basic and instrumental activities of daily living (1). Caring involves affective responsibility, zeal, bonding, and consideration for the history and concerns expressed by the older person, establishing a caregiver's commitment to promoting the wellbeing of the person cared for (2–4).

Caregivers are classified as formal or informal. Formal caregivers have had training and are paid for their services. Informal caregivers give care to family or friends without payment to perform this role (5). Family members are crucial to delivering long-term care for the older person; they often assume the role of caregivers without questioning their desire or aptitude for the activities (6, 7). Studies demonstrate that informal caregivers experience a significant burden in providing care to older adults with chronic illnesses (e.g., stroke, Parkinson's disease, dementia) or dependence (6, 8–13).

Caregiver burden refers to the multifaceted strain the caregiver perceives from caring for a family member and loved one over time (14). There are many interpretations of caregiver burden in the literature (14); among them, the most used is Pearlin's Stress Process (15, 16). In this model, the author has proposed a conceptual model of the burden by describing the impact of stressful situations on caregivers and emphasizing the presence of four domains that predispose to or minimize stress: (1) stress context, which involves social and economic characteristics of the caregiver; (2) stressful conditions, which are those that are anchored in the needs and demands of the older person as well as in the relationships between caregiver and older person; (3) stress mediators, which comprise conditions that can minimize negative repercussions of care such as coping strategies and social support; and (4) stress outcomes, which are manifestations of stress the physical and mental wellbeing of the caregiver (15).

Some conditions have been identified in the literature that affect the caregiving burden. These include dependence on the daily activities of the older person, providing care for long hours, having a lower level of education, conflicting older-caregiver relationships, living in the same house with the care recipient, being socially isolated, being under financial stress, and having no choice but to be a caregiver (10, 12, 17, 18). However, strengthening the support network and guidance on care are described as protective conditions that minimize the burden (14, 19).

The consequences of caregiver burden include negative repercussions to the caregiver care recipient. Burden threatens caregivers' physical, psychological, emotional, and functional health and may negatively affect their health and wellbeing. Caregivers can reduce care provisions and neglect the support for older adults when experiencing a burden (14).

In this scenario, it is necessary to clarify conditions that may relate to a burden among caregivers of older adult dependents. Professional health care has an essential impact on the health and wellbeing of caregivers and may be planning to train informal caregivers and support caregivers by aiding in care-related activities. Furthermore, it is necessary to identify caregiver burden and its related factors to facilitate health professionals in developing and implementing the appropriate interventions for caregiver burden prevention.

Therefore, this study aimed to investigate the relationship between burden, sociodemographic, caregiving, and health characteristics of informal caregivers of dependent older adults.

## 2. Materials and methods

A quantitative, cross-sectional, and analytical study was carried out in Palmas, Tocantins, Brazil.

The sample was calculated based on the following criteria: sample power of 0.95, mean effect size f = 0.50, a significance level of 5%, and an addition of 10% to the initial value for predicted losses, thus estimating the minimum number of 49 caregivers. The inclusion criteria were being a family member, aged 18 years or over, and caring for an older adult (≥ 60 years old) with a maximum need for care, and registered in a Family Health Strategy unit located in an urban area of the city (13). According to Nunes et al. (7), older persons with the maximum need for care need a caregiver to help full-time with bathing, toileting, personal hygiene, walking, and eating. Caregivers who, after three attempts, could not be contacted by telephone or located for the interview were excluded.

The interviews were realized at home with the community health agent after being scheduled by telephone. The interviewers applied a pre-tested, semi-structured questionnaire to collect sociodemographic and health information. The interviews were conducted between January 2020 and January 2022, with an average duration of 90 min. Due to the COVID-19 pandemic, data collections were suspended in March 2020 and resumed in October 2021.

Caregiver burden was assessed using the 22-item Zarit scale. Each item is scored on a scale from 0 to 4, with values of 0 (never), 1 (rarely), 2 (sometimes), 3 (often), and 4 (always). The scale scores range from 0 to 88 points, in which the higher the score, the greater the caregiver burden (20). The scale was validated by Brazilians, and care was considered a burden when the score was equal to or >21 points (21). The Zarit Burden Scale used in the present study obtained a Cronbach's alpha score of 0.84, indicating satisfactory reliability.

To understand the relationship between burden and other independent variables, these were described according to the caregiver stress model proposed by Pearlin et al. (15):

- *Stress context*: sex, age, education, marital status, monthly income, and cohabitation with the older person.

- *Stressors*: related to the provision of care (daily dedication to care, time of care, receiving help with caregiving) and family dysfunction.

The Family APGAR was used to assess family functioning from the perception and interaction between its members. The family APGAR scale is derived from a questionnaire to measure a subject's satisfaction with five components of family function: adaptation, partnership, growth, affection, and resolve. The items are scored from 0 to 2: always (2), sometimes (1), and never (0). Its score varies from 0 to 10; for analysis criteria, the sum ≤6 points was considered family dysfunction (22).

- *Stress mediators*: satisfaction with care and social support.

The Carer's Assessment of Satisfaction Index (CASI) assessed satisfaction with care, composed of 30 statements about positive aspects of caring. The higher the rating, the greater the caregiver satisfaction (23, 24). The CASI showed satisfactory reliability (Cronbach's alpha was 0.84).

Perceived social support was measured by the Medical Outcomes Study Social Support Survey (MOS), which is a 19-item survey that measures four dimensions of available support: material, affective, emotional, positive social interaction, and information. The score scales range from 20 to 100 points, in which the higher the score, the higher the level of social support (25). In this study, Cronbach's alpha coefficients of the types of social support varied between 0.76 and 0.87.

- *Stress manifestations*: depression symptoms assessed using the Patient Health Questionnaire-9 (PHQ-9).

The PQH-9 was valid for the Brazilian population and assessed nine symptoms as depressed mood, lack of energy, and changes in habits and life patterns (26). Caregivers with scores ≥5 were considered to have depressive symptoms. This scale presented good internal consistency in the present study (Cronbach's alpha was 0.77).

The software program Stata version 17 was used in the data analysis. The probability level ≤0.05 was used in all statistical tests. The variable caregiver burden was tested for normality using the Shapiro-Wilk test. The *t*-test was used to compare the means of burden and independent qualitative variables. The Pearson's correlation test was used to analyse the correlation between burden and quantitative independent variables. Multiple linear regression was used to analyse the associated factors that burden caregivers.

The Research Ethics Committee of the Federal University of Tocantins, opinion n°. 3.138.324/2019, approved the study. All participants signed the Free and Informed Consent Form after verbal and written explanations about the study.

## 3. Results

Fifty-two informal caregivers participated in the study. In the total sample, 38 (73.1%) were daughters, seven (13.5%) were spouses, three (5.8%) were grandchildren, two (3.8%) were sons-in-law, and two were (3.8%) siblings. Most older persons who received the care were women (73.1%), with a mean age of 79.3 years, and bedridden due to complications from chronic diseases or accidents by falls (93.3%). Stroke was the leading triggering cause of immobility in the older adults in this study (41.3%), followed by accidents due to falls (22.9%) and Alzheimer's dementia (14.6%).

Most informal caregivers were women (84.6%) under 60 years of age (80.8%). Most also stated having a partner (65.4%), reported family income less than or equal to one minimum wage (56.2%), reported having 12 years or more of schooling (53.8%), stated that they lived with the ol[d]der person (92.3%), and reported good family functionality (69.4%). As for caregiver burden, there was a mean score of 26.3 points (SD = 14.6; minimum = 0; maximum = 68) and a prevalence of 67.3%. No statistical relationship was detected between caregiver burden and demographic and socioeconomic characteristics (Table 1).

**Table 1:** Distribution of informal caregivers of older adults according to scores of burden and demographic, social, and economic characteristics. Palmas, Tocantins, Brazil (*n* = 52).

**Characteristic**	**Total *n* (%)**	**Caregiver burden**	
		**Mean (SD)**	***p*** **-value** ^*^
**Sex**			0.453
Male	8 (15.4)	26.9 (21.7)	
Female	44 (84.6)	26.2 (13.3)	
**Age group**			0.072
<60 years	42 (80.8)	27.8 (14.9)	
≥60 years	10 (19.2)	20.2 (12.3)	
**Marital status**			0.301
With partner	34 (65.4)	25.5 (14.5)	
Without partner	18 (34.6)	27.8 (15.1)	
**Schooling**			0.534
<12 years	24 (46.2)	26.5 (11.0)	
≥12 years	28 (53.8)	26.1 (17.4)	
**Family income (*****n*** **=** **32)**			0.384
≤ 1 minimum wage	18 (56.2)	25.0 (13.2)	
>1 minimum wage	14 (43.8)	23.4 (12.8)	
**Cohabits with older adult**			0.082
No	4 (7.7)	16.5 (5.6)	
Yes	48 (92.3)	27.1 (14.9)	
**Total**	52 (100.0)	26.3^**^ (14.6)	

* *p*-value from T-test;

** min = 0; max = 68.

The participants provided care >12 h a day (80.8%), received help (71.1%), and reported a time of care ≤4 years (51.9%). Burden scores were higher among caregivers who did not receive help from others in care (*p* = 0.016). One-third of caregivers (30.9%) reported family dysfunction, and among these, the mean burden scores were higher in those with good functionality (*p* = 0.001) (Table 2).

**Table 2:** Distribution of informal caregivers of older adults according to burdens, caregiving, family dysfunction, and depression symptoms. Palmas, Tocantins, Brazil (*n* = 52).

**Characteristic**	**Total *n* (%)**	**Burden caregiver**	
		**Mean (SD)**	*p*-value^*^
**Daily care time**			0.102
≤ 12 h	10 (19.2)	21.0 (19.1)	
>12 h	42 (80.8)	27.6 (13.3)	
**Get help from others**			0.016
No	15 (28.9)	33.1 (13.7)	
Yes	37 (71.1)	23.6 (14.2)	
**Care time**			0.136
≤ 4 years	27 (51.9)	24.1 (16.6)	
>4 years	25 (48.1)	28.6 (12.0)	
**Family dysfunction (*****n*** **=** **49)**			0.001
No	34 (69.4)	22.6 (12.4)	
Yes	15 (30.6)	36.7 (14.3)	
**Depression symptoms (*****n*** **=** **51)**			0.007
No	32 (62.7)	23.1 (12.5)	
Yes	19 (37.3)	33.2 (15.1)	
**Total**	52 (100.0)	26.3 (14.6)	

* *p*-value from T-test.

Table 2 also shows that some caregivers reported depressive symptoms (37.3%). Caregivers with depressive symptoms had higher mean burden scores than those without symptoms (*p* = 0.007).

There was a statistically significant positive difference between the caregiver burden and caregiving satisfaction scores (*r* = 0.76, *p* < 0.001). A significant negative correlation was found between the caregiver burden and material support (*r* = −0.30, *p* = 0.040) (Table 3).

**Table 3:** Correlation between caregiver burden, caregiving satisfaction, and social support. Palmas, Tocantins, Brazil. 2020–2022 (*n* = 52).

**Characteristics**	**Mean (SD)**	**Median (min-max)**	**Caregiver burden**	
			*r* ^*^	***p***
Caregiving satisfaction	53.4 (15.6)	52.0 (31.0–95.0)	0.76	**<0.001**
**Type of social support**				
Material	82.1 (25.5)	96.9 (20.0–100.0)	−0.30	**0.040**
Affective	88.3 (18.2)	100.0 (33.3–100.0)	−0.12	0.400
Emotional	73.3 (26.5)	81.3 (20.0–100.0)	−0.13	0.381
Information	70.4 (28.3)	81.3 (20.0–100.0)	−0.21	0.159
Positive social interaction	73.6 (27.4)	81.3 (20.0–100.0)	−0.28	0.053

* *p*-value from Pearson's correlation test.

In the adjusted regression model, satisfaction with care and family dysfunction were significantly associated with the burden score. Burden scores were 0.61 units higher for each unit of the care satisfaction score (β: 0.61, 95% CI: 0.40 to 0.82) and 8.07 units higher in those with family dysfunction (β: 8.07 95% CI: 0.69 to 15.44) (Table 4).

**Table 4:** Factors associated with the burden of caregivers. Palmas, Tocantins, Brazil, 2020–2022 (*n* = 52).

**Characteristics**	**β**	**95% CI**	***p*-value**
Caregiving satisfaction	0.61	0.40–0.82	<0.001
Family dysfunction (yes)	8.07	0.69–15.44	0.033
Help from others (yes)	−6.29	−13.30–0.73	0.077

R^2^ adjusted = 0.66.

## 4. Discussion

This study analyzed the relationship between burden, sociodemographic and caregiving characteristics, and health aspects of informal caregivers of dependent older adults. The results showed that the burden scores were higher among caregivers who did not receive help from others in care, who reported family dysfunction, and who had depression symptoms. A correlation was identified between scores of burdens and satisfaction with care and between burden and perceived material support.

These findings corroborate the theoretical model by Pearlin et al. (15), since receiving help and family dysfunction are considered stressful conditions. Satisfaction with care and perceived material support are stress mediators, while depression symptoms are stress manifestations.

In regard to the sample profile, in the assessment of the informal caregivers, most participants were women, adults, daughters, having low income, and living with an older person. These characteristics of the informal caregivers' profile are found in other studies in the same line of research (3, 5, 10, 11, 13, 18, 27, 28).

Families predominate among those responsible for providing care, and it is noteworthy that most informal caregivers are children of aging parents. Studies show that children take on this role as a form of compensation for care once received, and this reinforces the social role of the family in welcoming and caring for their sick family member (29). Studies on the issue of gender demonstrate the social and cultural belief that care is still a function assigned to women since they have an accumulation of responsibilities such as keeping the house, caring for their families, and non-household work when they manage to keep this bond (19).

Social and economic issues permeate this relationship and must be considered in comprehensive health care. The unfavorable financial condition because of changes in employment relationships and the lack of payment for the care provided makes the informal caregiver vulnerable. This situation can lead the caregiver to live in the same household as the older person. This family arrangement creates greater possibilities for caregiving support; however, the caregiver will be exposed to more burden, stress, and intergenerational conflicts (30).

In this study, family functionality was associated with the burden scores. Family functionality comprises how the family organizes itself in the face of the needs of family members, including older persons, while family dysfunction is considered a stressful condition between the members. The difficulty of family members in maintaining harmonious bonds can explain the relationship between family dysfunction and burden. A caregiver–older person relationship that is conflicting or insufficient affects the caregiver's physical, emotional, social, and economic conditions (18). Rico (29) points out that cohabitation can benefit the care recipient, as the informal caregiver will be accessible immediately. However, cohabitation harms the caregiver, bringing several essential losses, such as freedom and privacy. Consequently, the caregiver's leisure time and social life are restricted (2, 28).

Another stressor in caregiving is the absence of support from another person to provide care. Caregivers who do not count on the help of other people to perform care present higher scores of burdens. This condition may be explained because the caregivers taking care of other individuals for hours and long periods have absent or reduced healthy support networks or negative social interactions that accentuate the burden (31). Jawahir et al. (32) find that caregivers without help in care are more likely to have a health impact than those with assistance. This scenario points out a need for caregivers' support in providing care as public politics aid the demands of informal caregivers.

Regarding stress mediators, a negative correlation was identified between the scores of burdens and perceived material support. Material support refers to the availability of practical services and material resources, which include, for example, cash assistance or helps with household chores; therefore, this type of support can have a positive and buffering effect on the negative influence of caregiving on wellbeing during challenging caregiver tasks (33). It is essential to emphasize the relevance of this information when targeting these caregivers for future health interventions.

The results showed an association between satisfaction with care and burden scores. These findings can be explained by the tendency that the greater the burden, the greater the satisfaction for overcoming difficulties and the feeling of repayment of care to a family member. Another explication of these results may be due to the psychological resilience of caregivers. Psychological resilience is defined as the ability of a person to successfully overcome and adapt to adverse conditions despite difficult circumstances, and such resilience produces satisfaction with social networks and social support, psychological wellbeing, strength, and healthy life (34).

The literature points to the duality of perceptions about the act of caring, which are sometimes positive aspects and sometimes negative (27, 31, 35, 36). From the positive perspective, care is understood as satisfaction with the role of caring, involving emotional rewards, personal growth, development of skills and domains, strengthening and spiritual growth, expansion of relationships, and a sense of duty and reciprocity (3, 4). On the other hand, feelings of anguish, impatience, loneliness, frustration, anger, and sadness are mentioned as negative characteristics that emerge in the care routine (31, 36).

Emotional suffering triggers psychosomatic manifestations that can result in physical signs, which somatize and are physically expressed in the caregiver's body, as observed by Gomes et al. (19) in a study on the consequences of care for the health of senior caregivers of dependent family members.

The literature has highlighted the associated depressive symptoms and caregivers' burden (37–40). The presence of caregiving stress situations (e.g., functional status, cognitive impairment, and behavioral problems of the care recipient) does not cause direct depressive symptoms in the caregivers of older people. These symptoms originate from stress responses due to inefficient coping and are associated with high levels of psychological distress as clinical depression (39, 41).

Given the care burden experienced daily by informal caregivers at home, family engagement is essential, as is technical and psychosocial support by health professionals in the face of immersion in the care process experienced (30). Therefore, it is crucial to assess the caregiver burden to welcome and offer targeted and effective support for this population. In this respect, the Pan American Health Organization (42) proposed the following guidelines: assess the burden and stress of caring for dependent older adults, considering the proportion that the burden can assume in the life context of the person who becomes a caregiver; detect emotional changes that can trigger depression, anxiety, and a deterioration in self-care; identify the risk of older abuse; and offer support, through temporary assistance, guidance, training, financial aid, and psychological interventions.

Caring for those who provide care is challenging for professionals because it is necessary to identify several conditions predisposing to burden. Minimizing or reducing the caregiver burden is essential to support social health and monitor the quality of life and care given to older adults.

The study has limitations that need to be mentioned. The current findings were based on a sample from one county (Palmas) and assessed only caregivers of the dependent older adults residing in urban areas. Thus, the results may not be generalized to other locations and cultures. The nature of the study (a cross-section design) and the type of analysis limit the findings to associations rather than causal influences. However, in the present study, the scales used in the assessment of the conditions of caregivers were reliable, indicating satisfactory internal validity.

## 5. Conclusion

The findings of this study showed that caregivers with dysfunctional families and satisfaction with the care presented the highest-burden scores. Given this, there is a need to enable mediation and reduce the burden arising from care, either by strengthening the family support network or providing professional support. Health professionals should embrace caregivers in their challenges and guide them about care demands, seeking to mitigate the stress manifestations and promote a caregiving life with more quality for caregivers.

## Data availability statement

The original contributions presented in the study are included in the article/supplementary material, further inquiries can be directed to the corresponding author.

## Ethics statement

The studies involving human participants were reviewed and approved by the Research Ethics Committee of the Federal University of Tocantins, opinion no. 3,138,324/2019, approved the study. All participants signed the Free and Informed Consent Form after verbal and written explanations about the study. The patients/participants provided their written informed consent to participate in this study.

## Author contributions

JM and DN contributed equally to designing, constructing, applying questionnaires, data interpretation, and manuscript writing. IB and MG contributed equally to the application of questionnaires, interpretation, and data analysis. RN, AS-O, TB, VP, and MS contributed to the interpretation and analysis of data and writing the manuscript. SF, FM, and LC carried out a critical review of the manuscript. All authors approved the version submitted for publication.
